# Simultaneous determination of moxifloxacin and cefixime by first and ratio first derivative ultraviolet spectrophotometry

**DOI:** 10.1186/1752-153X-6-105

**Published:** 2012-09-20

**Authors:** Mahesh Attimarad, Bander E Al-Dhubiab, Ibrahim A Alhaider, Anroop B Nair, Harsha N Sree, Ahmed K Mueen

**Affiliations:** 1 Department of Pharmaceutical Sciences, College of Clinical Pharmacy, King Faisal University, Al-Ahsa, Saudi Arabia

**Keywords:** Moxifloxacin, Cefixime, Ratio first derivative UV, Spectroscopy, Validation

## Abstract

**Background:**

The new combination of moxifloxacin HCl and cefixime trihydrate is approved for the treatment of lower respiratory tract infections in adults. At initial formulation development and screening stage a fast and reliable method for the dissolution and release testing of moxifloxacin and cefixime were highly desirable. The zero order overlaid UV spectra of moxifloxacin and cefixime showed >90% overlapping. Hence, simple, accurate precise and validated two derivative spectrophotometric methods have been developed for the determination of moxifloxacin and cefixime.

**Methods:**

In the first derivative spectrophotometric method varying concentration of moxifloxacin and cefixime were prepared and scanned in the range of 200 to 400 nm and first derivative spectra were calculated (n = 1). The zero crossing wavelengths 287 nm and 317.9 nm were selected for determination of moxifloxacin and cefixime, respectively. In the second method the first derivative of ratio spectra was calculated and used for the determination of moxifloxacin and cefixime by measuring the peak intensity at 359.3 nm and 269.6 nm respectively.

**Results:**

Calibration graphs were established in the range of 1–16 μg /mL and 1–15 μg /mL for both the drugs by first and ratio first derivative spectroscopic methods respectively with good correlation coefficients. Average accuracy of assay of moxifloxacin and cefixime were found to be 100.68% and 98 93%, respectively. Relative standard deviations of both inter and intraday assays were less than 1.8%. Moreover, recovery of moxifloxacin and cefixime was more than 98.7% and 99.1%, respectively.

**Conclusions:**

The described derivative spectrophotometric methods are simple, rapid, accurate, precise and excellent alternative to sophisticated chromatographic techniques. Hence, the proposed methods can be used for the quality control of the cited drugs and can be extended for routine analysis of the drugs in formulations.

## Background

Ultraviolet (UV) – visible spectroscopic method of analysis is widely used in the analysis of drugs in pharmaceutical formulations and for dissolution and disintegration studies due to its good sensitivity and cost effectiveness. In last three decades, derivative spectrophotometry has been extensively used in the determination of drugs in multi components having overlapping spectra, which eliminates interference from formulation matrix by using the zero-crossing techniques [1-4]. Ratio-spectra derivative spectrophotometric method is another useful technique for the estimation of drugs in their mixtures [5-8].

The derivative spectra methods allows us to use the wavelength of highest value of maxima or minima signals. Moreover, the presence of a more number of maxima and minima wavelengths gives an opportunity to select a particular wavelength for determination of active compounds without the interference from other compounds or formulation excipients. The derivative spectra methods are developed for simultaneous determination of moxifloxacin Hydrochloride (MOX) and cefixime trihydrate (CEF), a newly FDA approved multicomponent formulation for combination therapy.

**Scheme 1:**
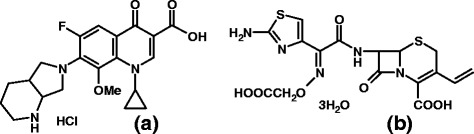
Structure of Moxifloxacin HCl (a) and Cefixime trihydrate (b).

Cefixime trihydrate [(6*R,*7*R*)-7-(2-(2-Amino-4-thiazolyl)glyoxylamido]-8-oxo-3-vinyl-5-thia-1-azabicyclo[4.2.0]oct-2-ene-2-carboxylic acid, 7^2^-(*Z*)-*O*-(carboxymethyl)oxime]trihydrate, Scheme 1) is semi synthetic, oral, third-generation cephalosporin antibiotic. Cefixime is active against a very wide spectrum of bacteria, act by inhibiting cell wall formation [9]. Literature reports many analytical methods for the determination of CEF in single and in combination with other drug, using UV spectroscopy [10,11] spectrofluorometry [12] HPLC [13-20] and HPTLC [21].

Moxifloxacin (1-cyclopropyl-7-(S, S)-2, 8-diazabicyclo (4.3.0)-non-8-yl-6-fluoro-8-methoxy-1,4-dihydro-4-oxo-3-quinoline carboxylic acid hydrochloride, MOX, Scheme 1) is antimicrobial agent, it is fourth generation fluoroquinolone antibiotic. The mechanism of action involve inhibition of an enzyme topoisomerase II (DNA gyrase), which is essential for bacterial DNA replication [22]. Several analytical methods have been reported for the determination of MOX in formulations and biological fluids, such as UV spectroscopic methods [23,24], Spectrofluorometry [25], RP-HPLC [26-30], and capillary electrophoresis [31,32].

The new combination of MOX and CEF is approved by Central Drugs Standard Control Organization (CDSCO) India for the treatment of lower respiratory tract infections in adults. Simultaneous determination of these drugs is essential in each step of initial formulation development and screening stage of any solid dosage form. A fast and reliable method for the dissolution and release testing of MOX and CEF were highly desirable. However, there are no simple and rapid analytical methods to estimate the drug content in the combined forms. Hence there is an urgent demand to develop a simple and rapid method such as spectroscopic method to assess the drug content in this combination. However, the major concern for these two drugs is the overlapping of absorption bands, which also restricts the direct measurement using UV spectroscopy. The objective of the current study was to develop rapid, accurate, reproducible, validated and economical first derivative and ratio first derivative analytical methods for the simultaneous determination of MOX and CEF from pure and in presence of formulation excipients.

### Experimental

#### Instruments and chemicals

UV-visible spectrophotometer (Shimadzu, 1700) with UV-probe software connected to computer was used for the drug estimation. Quartz cuvettes (1 cm) were matched and used for all absorbance measurements. Double distilled de-ionized water and Whatmann filter paper (no.41) were used throughout the experimental work. The sample of moxifloxacin certified to contain 99.81% was procured from Micro Labs, India and Cefixime trihydrate certified to contain 99.84% from Dr. Reddy's laboratory, India. Moxicip tablets containing 400 mg of moxifloxacin and suprax tablets containing 400 mg of cefixime were purchased from pharmacy.

#### Preparation of standard stock solution

Standard stock solutions of MOX and CEF (1 mg/mL) were prepared by dissolving 100 mg of drugs in water, separately. The working standard solutions of the respective drugs were prepared by serial dilution using water.

#### Method development

##### Method-D1 (First derivative Spectrophotometric method)

In the first order derivative method, aliquots of MOX and CEF standard stock solutions were accurately transferred in to 10 mL volumetric flasks, separately and volumes were completed with water. All the solutions were scanned from 200 to 400 nm in the spectrum mode. Thus obtained absorption spectra were derivatized from first to fourth order. First order derivative (n = 1) spectra showed good sensitivity and linearity hence the zero crossing wavelengths, 287 nm and 317.9 nm of first order derivative spectra were selected for analysis of MOX and CEF, respectively. The calibration curves were constructed and the concentration of individual drug present in the mixture was determined against calibration curve in quantitation mode.

##### Method-RD1 (First derivative of the ratio spectra)

Previously scanned absorption spectra of MOX solutions prepared at different concentrations (1 to 15 μg/mL) in its binary mixture with CEF was divided by the spectrum of the standard solution of CEF (10 μg /mL in water) to get the ratio spectra of MOX. The first derivative of the ratio spectra were than calculated. The amount of MOX was determined by measuring the first derivative signal at 359.3 nm. A similar procedure was followed for different concentrations of CEF (1 to 15 μg /mL ) with MOX and for division spectrum of the standard solution of MOX (8 μg /mL in water) was used. Similarly, content of CEF was determined by measuring the first derivative signal at 269.6 nm.

##### Assay of laboratory-prepared mixtures

The absorption spectrum was recorded for the laboratory prepared mixtures, against water as a blank. The intensity of the first derivative spectra of the laboratory prepared mixtures containing different ratios of MOX and CEF were measured at 287 nm and 317.9 nm respectively. The previously scanned zero order absorption spectra for the laboratory prepared mixture were divided by the spectrum of CEF (10 μg/mL) and by spectrum of MOX (8 μg/mL) separately for the determination of MOX and CEF respectively. The concentrations of MOX and CEF were calculated from their corresponding regression equations measuring the intensity of signals at 359.3 nm and 269.6 nm respectively.

##### Analysis of MOX and CEF in presence of tablet excipients

To determine the content of MOX and CEF simultaneously in the presence of tablet excipients 20 tablets of MOX 400 mg and 20 tablets of CEF 400 mg were weighed separately and average weight was determined. All the 40 tablets were powdered and correct amount of powder (equivalent to 20 mg of both the drugs) was dissolved in water using sonicater (30 min). After filtration, an appropriate aliquots were subjected to above methods and the amount of MOX and CEF were determined.

##### Validation of the methods

Newly developed methods were validated for linearity, accuracy, precision, limits of quantization and selectivity according to the ICH guidance.

##### Linearity

For linearity six different concentration of MOX and CEF in the range of 1–16 μg/mL were analyzed. The limit of detection (LOD) and limit of quantification (LOQ) were determined by 3.3 σ/s and 10 σ/s criteria, respectively; where σ is the standard deviation of the analytical signal and s is the slope of the corresponding calibration curve.

##### Accuracy

Accuracy of the method was determined by calculating recoveries of MOX and CEF by standard addition method, in which pre-analyzed samples were taken (5 μg/mL ) and standard drug was added at three different levels i.e. 80%, 100% and 120%. The total amount of MOX and CEF were estimated by using the proposed methods in triplicate. The% recovery of the added pure drugs were calculated as% recovery = ((Dt-Ds)/Da) X 100, where Dt is the total drug concentration measured after standard addition; Ds drug concentration in the formulation mixture; Da drug concentration added.

##### Precision

Intraday and interday precision of the proposed methods were determined by estimating the MOX and CEF three times on the same day to obtain the repeatability and on three different days to obtain the reproducibility. Each assay was carried out on different samples of MOX and CEF. From the obtained data percent relative standard deviation (% RSD) was calculated.

##### Stability of the analytical solutions

This was evaluated by measuring absorbance of freshly prepared standard and sample solutions, and repeating the measurement after 24 h.

## Results and discussion

### Method development and validity

**Figure 1:**
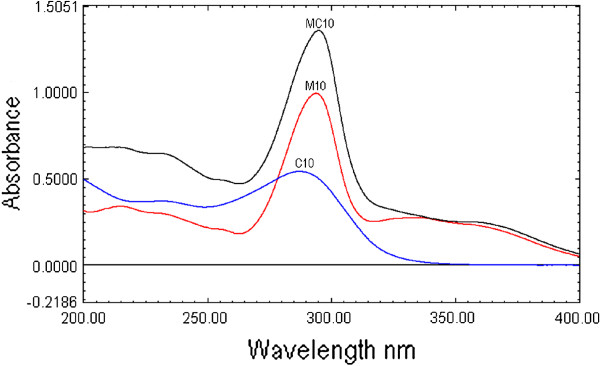
UV absorption spectra of Moxifloxacin (10 μg/mL, M10), cefixime (10 μg/mL, C10) and combination (10 μg/mL each, MC10).

MOX and CEF possess good aqueous solubility and showed good UV absorption, thus water has been selected as solvent for the present analytical methods. The zero order overlaid UV spectra of MOX and CEF (10 μg/mL each) and their mixture obtained were shown in Figure 1. It is evident from the figure that >90% of spectra are overlapping each other, demonstrating the complexity in measuring these drugs by direct UV absorption measurement in a binary mixture.

From the zero-order spectra first to four orders derivative spectra were obtained using digital differentiation for both the drugs. First order derivative spectra of both the drugs showed well-defined zones for determination of each analyte with a good sensitivity and linearity. Spectra with higher order of derivation had lower sensitivity and linearity, hence, only first order derivative spectra were selected for quantitative analysis. For first derivative spectroscopy, the spectra of the MOX and CEF obtained by scanning in water, were changed to first derivative spectra. For the first derivative spectra, different wavelengths (2, 4, 8 and 10 nm) were attempted to obtain the optimum Δλ. The results signified that 4 nm is an optimum Δλ and this wavelength was selected and used.

**Figure 2:**
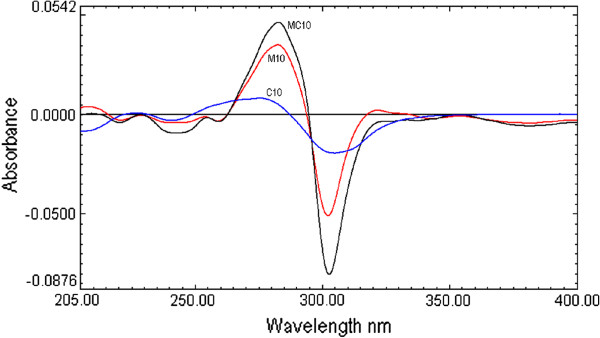
First derivative UV absorption spectra of moxifloxacin (10 μg/mL, M10), cefixime (10 μg/mL, C10) and combination (10 μg/mL each MC10).

**Figure 3:**
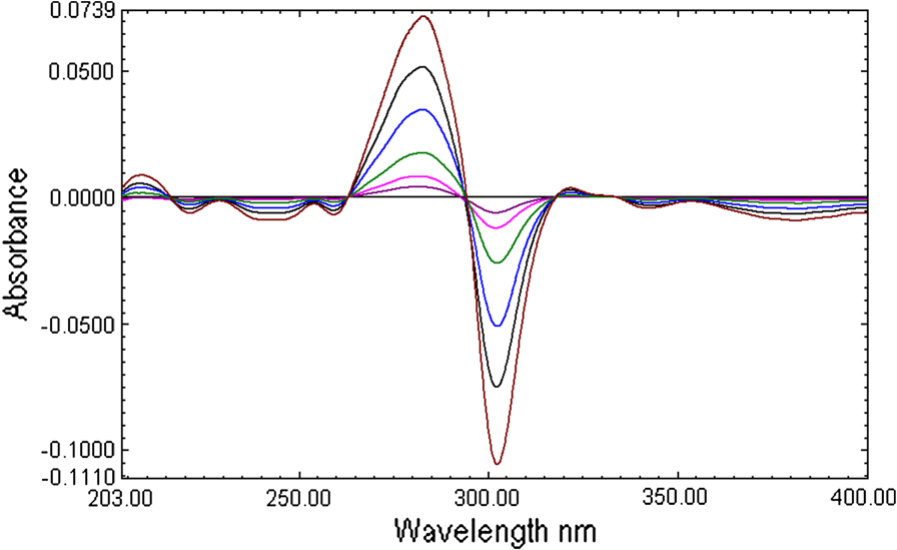
First derivative UV absorption spectra of Moxifloxacin (1 to 16 μg/mL).

**Figure 4:**
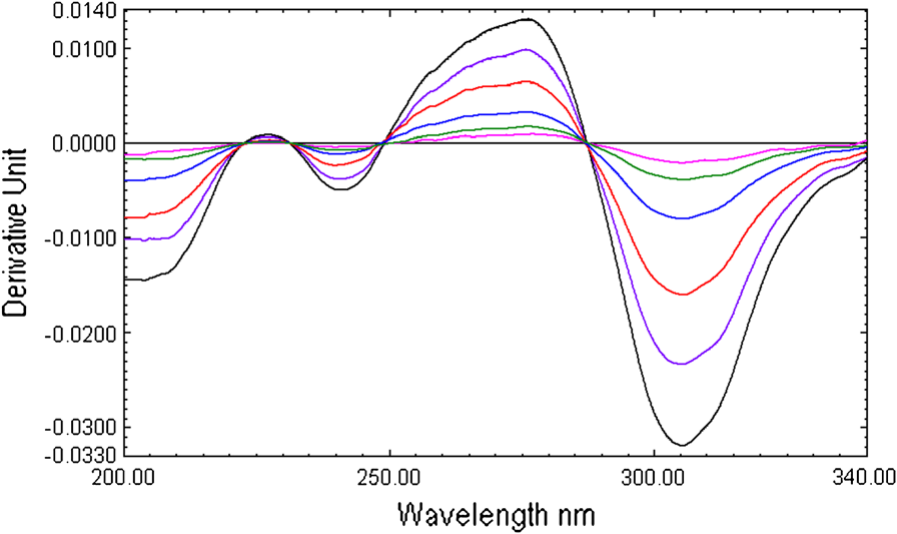
First derivative UV absorption spectra of cefixime (1 to 16 μg/mL).

The first derivative spectra of CEF present four zero crossing at 222.1, 248.4, 379.0 nm and 287.0 nm (Figure 2 and Figure 3). Therefore, to select most appropriate wavelength, MOX was determined form the laboratory mixture at all the four wavelength and mean recovery and standard deviation were calculated and found to be 101 ± 2.45, 100.89 ±1.48 99.81 ± 2.68 and 100.67 ± 0.73 at 222.1, 248.2, 379.0 nm and 287.0 nm respectively. Further, the first derivative spectra of MOX showed two zero crossing wavelengths (317.9 nm and 333.7 nm) (Figure 2 and Figure 4). These points were tested for the determination of CEF from the binary mixture. The synthetic mixture mean recovery of CEF and standard deviation were found to be 99.5 ± 1.06 and 101.85 ±1.92 at 317.9 and 333.7 nm respectively. The 287.0 nm peak for MOX and 317.9 nm peak for CEF were selected for determination of drug in synthetic mixture in pure and in presence of tablet excipients due to low standard deviation and good mean recovery. Moreover, the method was linear in the concentration range of 1–16 μg /mL for both MOX and CEF with correlation coefficient 0.9992.

**Figure 5:**
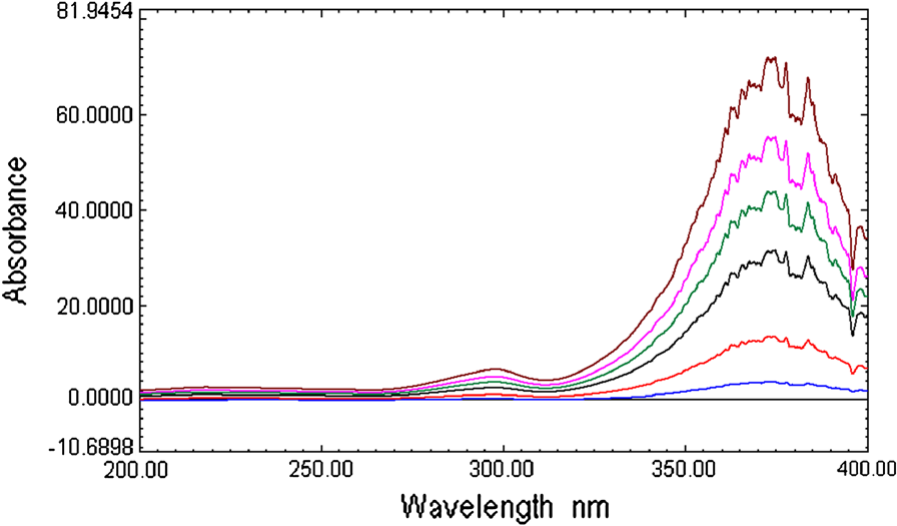
Ratio spectra of MOX (1 to 15 μg/mL) using 10 μg/mLsolution of CEF as devisor.

**Figure 6:**
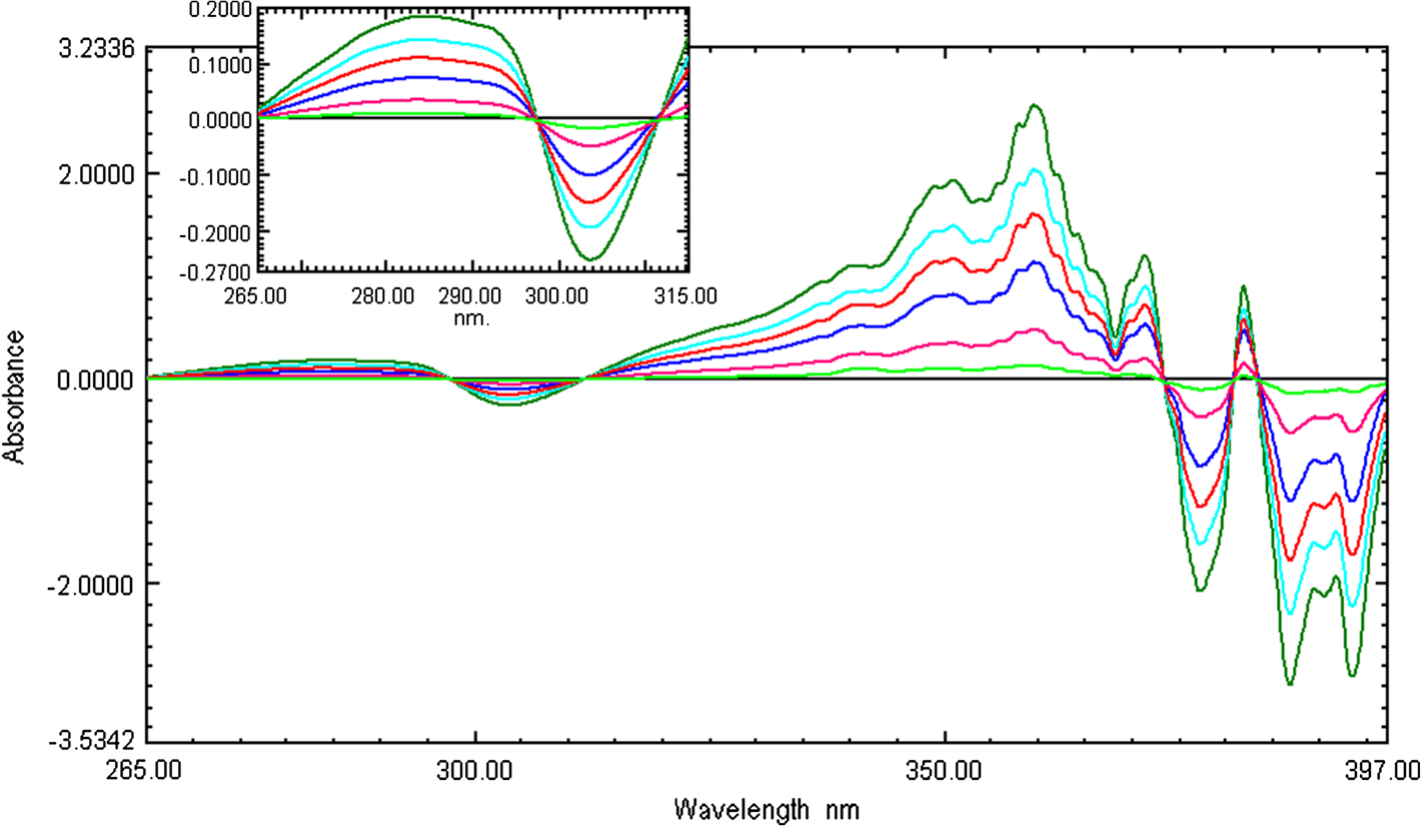
First derivative Ratio spectra of MOX (1 to 15 μg/mL), inside enlarged portion of spectra of 265 to 315 nm.

**Figure 7:**
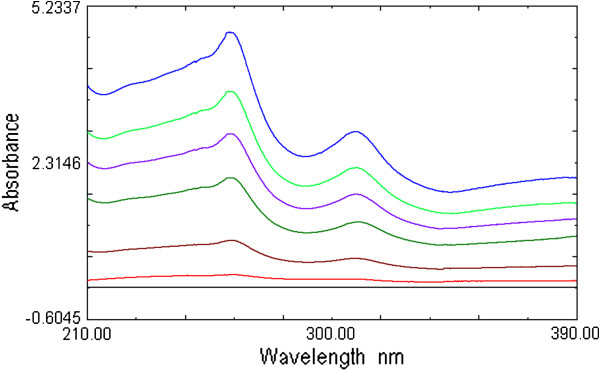
Ratio spectra of CEF (1 to 15 μg/mL) using 8 μg/mL solution of MOX as devisor.

**Figure 8:**
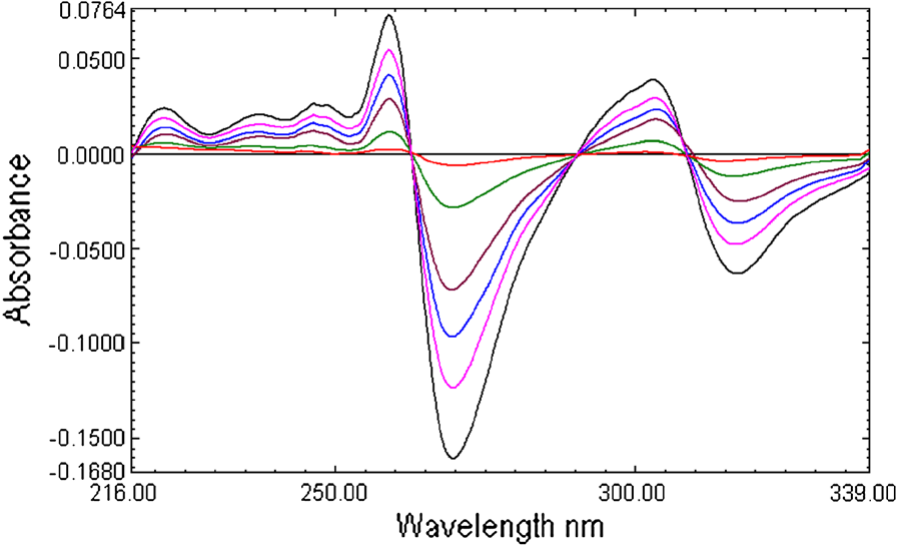
First derivative of the ratio spectra of CEF (1 to 15 μg /mL).

The selection of the divisor concentration and the working wavelengths were very important, hence, eight different concentrations of CEF and MOX (2, 4, 6 … and 16 μg /mL) were tried as divisors separately. It was found that minimum noise and better selectivity were obtained upon using 10 μg/mL of CEF spectrum and 8 μg /mL of MOX spectrum as a divisor. For the determination of MOX, the UV spectra of MOX and CEF standards of increasing concentration were divided by the spectrum of 10 μg /mL CEF solution, from the spectra obtained (Figure 5) first derivative spectra were calculated. It is evident from the figure (Figure 6) that there are two maxima at 283.5 and 359.3 nm and four minima at 303.5, 377.1, 386.8 and 393.4 nm. It was found that measured signals at these wavelengths are proportional to the concentrations of the drug. Similarly, for determination of CEF, UV spectra of CEF standards of increasing concentrations in its binary mixture with MOX were divided by the spectrum of 8 μg /mL MOX. From the ratio spectra obtained (Figure 7), their first derivatives were calculated (Figure 8). These spectra showed three maxima (221.3, 258.9 and 303.2 nm) and two minima (269.6 and 316.3 nm). Further, the results also indicated that the measured signals at these wavelengths are proportional to the concentrations of the drug. The wavelength of 359.3 and 269.6 nm were selected for determination of MOX and CEF in synthetic mixture and in the presence of tablet excipients due to its low standard deviation value and suitable mean recovery at these wavelengths.

### Linearity

Calibration graphs were established for MOX and CEF in the concentration range 1 to 16 μg /mL at 287.0 nm for MOX and at 317.9 nm for CEF by first derivative method. Whereas by the first derivative of the ratio spectrum calibration curves were constructed at 359.3 nm for MOX and 269.6 nm for CEF in the range of 1 to 15 μg /mL . The linearity of the calibration curves were validated by the high value of correlation coefficients. The analytical data of the calibration curves including the slope and intercept are summarized in Tables. The proposed methods were validated as per ICH guideline and the limit of detection (LOD) and limit of Quantization (LOQ) for method D1 and method RD1 for both the drugs were calculated (Table 1) and tested practically.

**Table 1:** Validation parameters for first and ratio first derivative spectroscopic methods

** Parameters assessed**	Method D1*		Method RD1**	
	**MOX**	**CEF**	**MOX**	**CEF**
Beer’s law range (μg /mL )	1-16	1-16	1-15	1-15
Wavelength (nm)	287.0	317.9	359.3	269.6
Correlation Coefficent(r^2^)	0.9992	0.9992	0.9991	0.9993
Slope	0.00362	0.00114	0.1807	0.0109
Intercept	5.08x10^-4^	−4.32x10^-5^	−0.0196	−0.0016
LOD	0.22	0.32	0.24	0.14
LOQ	0.75	0.95	0.70	0.43
Intraday precision (%RSD)	0.87	1.35	0.92	0.80
Interday precision(%RSD)	1.56	1.79	1.38	1.18

* First derivative Spectrophotometric method. **First derivative of the ratio spectra.

MOX-Moxifloxacin HCl, CEF- Cefixime trihydrate. %*RSD* : Percent Relative Standard Deviation.

### Precision

Relatively very low % RSD for inter and intraday variations for both drugs in both methods confirms the precision of the methods (Table 1). RSD values found were well with the acceptable range indicating that these methods have excellent repeatability and reproducibility in the current experimental condition.

### Accuracy

The results showed that the % recovery values are greater than 98% with low standard deviation indicating high accuracy of the proposed analytical methods. Further the validity and reliability of the proposed methods were assessed by determining the mean percentage recovery at 80%, 100% and 120% level. The average % recovery ranges from 98.72 to 100.57 for MOX and CEF form both the methods and are presented in Table 2.

**Table 2:** Recovery studies

Amount of Base concentration (μg /mL )*		**Amount of Pure Drug Added (μg /mL )**		**% Recovery**			
				**Method D1**		**Method RD1**	
**MOX**	**CEF**	**MOX**	**CEF**	**MOX**	**CEF**	**MOX**	**CEF**
5	5	4	4	101.29	99.01	98.28	99.23
5	5	5	5	98.34	100.21	100. 81	101.18
5	5	6	6	101.38	98.28	99.15	101.29
		Average		100.34	99.17	98.72	100.57
		± RSD (%)		1.72	0.98	0.62	1.15

*Amount of MOX and CEF in the pre-analyzed samples is 5 μg/mL.

### Estimation in pure and in presence of tablet excipients

For quantitative analysis of MOX and CEF laboratory mixed pure drugs and solid dosage formulation were used and analyzed by first and ratio first derivative methods. Results showed that the% amount of MOX and CEF was found to be between 98.93 to 101.32 with low standard deviation (Table 3).

**Table 3:** Results of Assay of formulation by first and ratio first derivative spectroscopic methods

**Weight taken (mg each)**	**% Amount found**			
	**Method D1**		**Method RD1**	
	**MOX**	**CEF**	**MOX**	**CEF**
Laboratory Mixture				
20.1	101.82	101.75	99.13	99.62
20	101.35	100.89	101.56	98.92
20	98.88	101.31	100.71	98.25
Avg.	100.86	101.32	100.47	98.93
± RSD (%)	1.58	0.42	1.22	0.69
Tablet powder				
20.1	100.4	99.12	99.01	101.13
20.1	101.74	98.95	98.91	101.01
20.2	101.12	101.7	101.61	98.56
Avg.	101.09	99.92	99.84	100.23
± RSD (%)	0.65	1.54	1.53	1.44

### Specificity

Since information of placebo composition is not available, the comparative study was carried out for laboratory mixed standards and the formulation under investigation. When comparing the results (Table 3) of laboratory mixed and the formulation preparation one can conclude that the method is specific for the analyte used and the coexisting constituents do not affect the measurements.

### Stability of the analytical solutions

Deviation from the mean initial absorbance is less than 2.4% for both MOX and CEF, which is well within the acceptable range (not more than ± 3%) indicating the stability of the analytical solutions.

## Conclusions

A simple, rapid, accurate, and precise and nature friendly two derivative UV spectroscopic methods were developed for the simultaneous estimation of MOX and CEF in bulk drugs and in the presence of tablet excipients. The recovery studies suggested non-interference of formulations excipients in the estimation. Moreover, the present methods were rapid as compared to sophisticated chromatographic techniques, hence the proposed methods can be used for the quality control of the cited drugs and can be extended for routine analysis of the drugs in their pharmaceutical preparations.

## Competing interests

The authors declare that they have no competing interests.

## Authors’ contributions

MA designed the proposed method. BEA proposed, planned and supervised the whole work. IAA coordinated the study and drafting the manuscript. ABN analyzed the data statistically and revised manuscript critically. SHN and MA carried out the experimental work. MAK helped in stability studies. All authors read and approved the final manuscript.
